# Error Analysis of the K-Rb-^21^Ne Comagnetometer Space-Stable Inertial Navigation System

**DOI:** 10.3390/s18020670

**Published:** 2018-02-24

**Authors:** Qingzhong Cai, Gongliu Yang, Wei Quan, Ningfang Song, Yongqiang Tu, Yiliang Liu

**Affiliations:** 1 School of Instrument Science and Opto-electronics Engineering, Beihang University, Beijing 100191, China; bhu17-yang@139.com (G.Y.); quanwei@buaa.edu.cn (W.Q.); songnf@buaa.edu.cn (N.S.); tuyq_1992@163.com (Y.T.); 2 National Remote Sensing Center of China, Beijing 100036, China; yiliang_liu91@163.com

**Keywords:** inertial navigation, comagnetometer, space-stable inertial navigation system, error analysis

## Abstract

According to the application characteristics of the K-Rb-^21^Ne comagnetometer, a space-stable navigation mechanization is designed and the requirements of the comagnetometer prototype are presented. By analysing the error propagation rule of the space-stable Inertial Navigation System (INS), the three biases, the scale factor of the *z*-axis, and the misalignment of the *x*- and *y*-axis non-orthogonal with the *z*-axis, are confirmed to be the main error source. A numerical simulation of the mathematical model for each single error verified the theoretical analysis result of the system’s error propagation rule. Thus, numerical simulation based on the semi-physical data result proves the feasibility of the navigation scheme proposed in this paper.

## 1. Introduction

With the development of quantum physics, the atomic gyroscope, based on quantum sensing technology, has gained widespread attention. The Inertial Navigation System (INS) with atomic gyroscopes has great potential in long-term independent navigation applications, such as underwater deep-sea robots [1,2]. There are two types of atomic gyroscope in theory [3]: Atomic Interferometry Gyroscopes (AIGs) [4,5,6,7], and Atomic Spin Gyroscopes (ASGs) [8,9,10,11,12,13,14,15]. The ASG does not need much laser power and linewidth, has no need for high vacuum, and is insensitive to gravity. As a result, it has been widely researched in recent years. The research on ASG has transitioned from the theoretical research phase to the prototype development and experimenting phase [16,17,18,19]. Fang et al. deduced the dynamics equation of the ASG [20]. The random drift of the atomic spin gyroscope was modelled and optimised by Quan et al. [21], Duan et al. [22], and Zou et al. [23]. The scale factor of the ASG was calibrated by Zou et al. [24]. The cross-talk effect of a dual-axis ASG was suppressed by Jiang et al. [25].

Based on the atomic Spin-Exchange Relaxation Free (SERF), the hybrid optical pumping can measure with high sensitivity the magnetic field or angular velocity [26,27,28]. Significant breakthroughs in the research of the K-Rb-^21^Ne comagnetometer prototype have provided a base for studying comagnetometer navigation methods. Because the angular velocity measurement is based on the spin atomic’s inertia and precession, it has a small measuring range just like the mechanical gyroscope does, the signal detection is performed using linear polarised light, which causes the comagnetometer to not be torqued on as an optical gyroscope. Thus, the comagnetometer cannot be used in either a traditional strap-down INS or a north-slaved local-level platform INS. Thus, it is necessary to carry out theoretical research on the comagnetometer INS, verify its principle and navigation accuracy to promote comagnetometer optimal design, and analyse the early stage requirements of the comagnetometer.

The comagnetometer INS is aimed at long-term, high-accuracy independent navigation for the submarine. The comagnetometer is similar to the electrostatic gyroscope (ESG), and thus can adopt a space-stable platform system as its navigation scheme [29,30,31,32]. The gyroscope works in a space-stable inertial frame when using a space-stable platform that is suitable for the comagnetometer INS, because it is conducive to the gyroscope’s optimal accuracy without a large measuring range or torqueing to the gyroscope. Existing research results on space-stable INS mainly apply to missile navigation systems [33,34,35,36], which does not apply to the long-term navigation of the comagnetometer INS. Thus, this paper aims to design a special space-stable navigation scheme for the comagnetometer INS, present the requirements of the comagnetometer prototype, and analyse the theoretical accuracy by determining the error propagation function.

This paper is organised as follows. In section two, the structure of a space-stable platform and its requirements of the comagnetometer are discussed. In section three, the mechanization equation of the comagnetometer space-stable INS is introduced. In section four, the theoretical accuracy of the comagnetometer INS is analysed. In section five, the semi-physical simulation results are presented as well as the accuracy verification. The last section concludes and discusses the findings.

## 2. Characteristic and Error Model of the Comagnetometer

### 2.1. Characteristic

The dynamics of the comagnetometer can be described by Bloch equations [3,16,20]. In steady state, keeping the leading order terms of each magnetic field and light shift, if the pump beam is in the z-direction and the probe beams are in the *x* and *y* direction, the Bloch equations are [16]:

(1) $$\begin{array}{l} P_{x}^{e}=\frac{P_{z}^{e}\gamma_{e}}{R_{tot}^{e}}\cdot \frac{\Omega_{y}}{\gamma_{n}}\\ P_{y}^{e}=\frac{P_{z}^{e}\gamma_{e}}{R_{tot}^{e}}\cdot \frac{\Omega_{x}}{\gamma_{n}} \end{array}$$

where, $P_{x}^{e}$, $P_{y}^{e}$ and $P_{z}^{e}$ are the polarization of electron spin of the alkali metal atom in *x*-, *y*- and z-direction; $\gamma_{e}$ is the electron gyromagnetic ratio and $\gamma_{n}$ is the nuclear gyromagnetic ratio; $R_{tot}^{e}$ is the total relaxation rate of electron spin. $\Omega_{x}$ and $\Omega_{y}$ are the measurement rotation in *x*- and *y*-direction.

Since the probe beam in *x*-direction is used to detect $P_{x}^{e}$, it can sense the rotation in *y*-direction. Similarly, the probe beam in *y*-direction is used to detect $P_{y}^{e}$ and senses the rotation in *x*-direction. If both probe beams are used in ASG, the ASG is actually a dual-axis gyroscope [17].

The transfer function of the comagnetometer deduced by the Bloch equations can be simplified as [20]:

(2) $$G\left(s\right)=\frac{k\omega_{n}^{2}}{s^{2}-2\zeta \omega_{n}s+\omega_{n}^{2}}$$

where, k, ζ and $\omega_{n}$ are comprised of ASG-related parameters.

It can be seen from Equations (1) and (2) that the transfer function of the comagnetometer is similar to a dual-axis rate gyroscope. As previously mentioned, the angular velocity measurement is based on the spin atomic inertia and precession, similar to the mechanical gyroscope.

According to the measurement principle of the comagnetometer, the nuclear spin angular momentum (NSAM) helps the electron spin angular momentum (ESAM) to avoid being affected by the magnetic field, but the strength of the compensation field generated by the NSAM is limited to a quick automatic compensation of the magnetic field [1]. The measuring range cannot be very large—with an experimental value of ±5°/s on the existing prototype—thus, the comagnetometer does not apply to a strap-down INS like the traditional optical gyroscope does.

The signal detection of the comagnetometer is based on linear polarised light detection, which is very similar to the optical gyroscope. Regarding the torqueing condition, because the comagnetometer’s rotor consists of innumerable atomics, which is different from a traditional mechanic rotor, high accuracy torqueing cannot be exerted on spin atomic like a mechanical gyroscope does. Therefore, it is similar to an optical gyroscope in that the comagnetometer does not apply to a traditional north-slaved local-level platform INS, which requires torqueing on the gyroscope.

### 2.2. Error Model

Taking the interference factors of magnetic interference and light into account, the leading terms of transverse electron spin polarization can be simply expressed as [25]:

(3) $$\begin{array}{l} P_{x}^{e}=\frac{-\gamma_{e}P_{z}^{e}R_{tot}^{e}}{D_{s}}\cdot \left[\frac{\Omega_{y}}{\gamma }+\frac{\gamma_{e}\Omega_{x}}{\gamma R_{tot}^{e}}\left(\delta B_{z}+L_{z}\right)-\left(\delta B_{z}+L_{z}\right)\gamma_{e}L_{x}-R_{tot}^{e}L_{y}\right]\\ P_{y}^{e}=\frac{\gamma_{e}P_{z}^{e}R_{tot}^{e}}{D_{s}}\cdot \left[\frac{\Omega_{x}}{\gamma }-\frac{\gamma_{e}\Omega_{y}}{\gamma R_{tot}^{e}}\left(\delta B_{z}+L_{z}\right)-R_{tot}^{e}L_{x}+\left(\delta B_{z}+L_{z}\right)\gamma_{e}L_{y}\right] \end{array},$$

where, $D_{s}=R_{tot}^{e}{}^{2}+\gamma_{e}{\left(\delta B_{z}+L_{z}\right)}^{2}$, $\gamma =\gamma_{e}\gamma_{n}/\left(\gamma_{e}-Q\gamma_{n}\right)$, Q is the electron slowing-down factor due to hopping between hyperfine levels, $\delta B_{z}$ is the magnetic interference in *z*-axis direction, $L_{x}$, $L_{y}$ and $L_{z}$ are the light shifts in the *x*-, *y*,- and *z*-axis direction.

The error model can be transformed as

(4) $$\begin{array}{l} P_{x}^{e}=S_{y}\Omega_{y}+\alpha_{yx}\Omega_{x}+\varepsilon_{y}\\ P_{y}^{e}=S_{x}\Omega_{x}+\alpha_{xy}\Omega_{y}+\varepsilon_{x} \end{array}$$

where, $S_{x}=\frac{\gamma_{e}P_{z}^{e}R_{tot}^{e}}{\gamma D_{s}}$ and $S_{y}=-\frac{\gamma_{e}P_{z}^{e}R_{tot}^{e}}{\gamma D_{s}}$ are the scale factor, $\alpha_{xy}=-\frac{\gamma_{e}{}^{2}P_{z}^{e}}{\gamma D_{s}}\left(\delta B_{z}+L_{z}\right)$ and $\alpha_{yx}=-\frac{\gamma_{e}{}^{2}P_{z}^{e}R_{tot}^{e}}{\gamma D_{s}}\left(\delta B_{z}+L_{z}\right)$ are the misalignment, $\varepsilon_{x}=-\frac{\gamma_{e}P_{z}^{e}R_{tot}^{e}{}^{2}}{D_{s}}L_{x}+\frac{\gamma_{e}{}^{2}P_{z}^{e}R_{tot}^{e}\left(\delta B_{z}+L_{z}\right)}{D_{s}}L_{y}$ and $\varepsilon_{y}=\frac{\gamma_{e}{}^{2}P_{z}^{e}R_{tot}^{e}\left(\delta B_{z}+L_{z}\right)}{D_{s}}L_{x}+\frac{\gamma_{e}P_{z}^{e}R_{tot}^{e}{}^{2}}{D_{s}}L_{y}$ are the bias of comagnetometer.

## 3. Comagnetometer Space-Stable Platform

### 3.1. Structure of the Space-Stable Platform

As explained above, the application characteristic of the comagnetometer does not match the scheme of the widely researched north-slaved, local-level platform INS, or the strap-down INS. The space-stable INS adopts a space-stable platform instead of exerting precessional torque on the gyroscope. On the space-stable platform, the gyroscope can operate freely to help the comagnetometer reach the optimal accuracy. Thus, the space-stable platform navigation scheme is considered.

**Figure 1:**
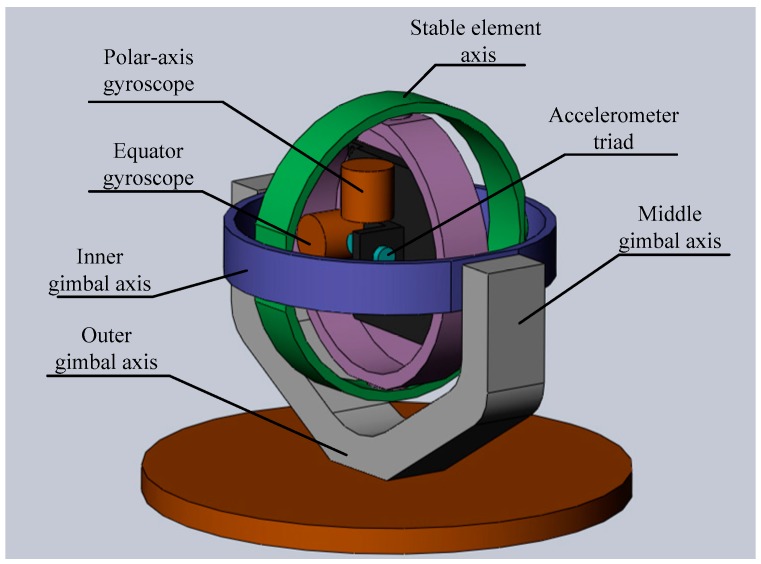
Structure of the space-stable platform.

The structure of the space-stable platform is shown in Figure 1. The platform is composed of the stable element, inner gimbal, middle gimbal, and outer gimbal from the inside out. Two dual-axis comagnetometers and an accelerometer triad are installed on the stable element. One comagnetometer has an atomic spin direction parallel to the polar-axis (called a polar-axis gyroscope), and the other one has an atomic spin direction parallel to the equator (called the equator gyroscope). The polar-axis gyroscope produces a two-degree-of-freedom angle, or angular velocity signals, to control the inner and middle gimbal axis. The equator gyroscope produces a one-degree-of-freedom angle, or angular velocity signals, to control the stable element axis. The outer gimbal axis is taken as a redundant axis controlled by the angle sensor output signal on the inner gimbal axis, or stable element axis; it is used to avoid a gimbal lock when the three axes are located in the same plane. The comagnetometers are not torqued on and the stable element functions in a space-stable state.

### 3.2. Discussion of the Requirements for the Comagnetometer Prototype

At present, the comagnetometer prototype operates in an angular velocity measurement mode based on gyroscopic precession. There are two problems that apply to the servo control of the space-stable platform of the comagnetometer that must be solved. One problem is dynamic performance, including the damping factor and the bandwidth. Although the sampling rate is set higher, the bandwidth of the comagnetometer prototype cannot be higher than 10 Hz, which cannot ensure the stability of the platform; yet according to the design experience of the platform INS, it requires a bandwidth higher than 100 Hz. The other problem is measurement error accumulation. Because the platform is controlled by the angular velocity signal, the attitude error of the platform accumulates during the whole navigation process. It requires the comagnetometer to have a much higher stability.

In the future, the comagnetometer prototype can be improved and will operate in an angle measurement mode based on gyroscopic inertia. As the signal can be detected by linearly polarised light, the measuring frequency will be high enough (1 kHz) to ensure the platform’s stability.

## 4. Error Analysis of the Comagnetometer Space-Stable INS

### 4.1. Mechanization Equation

Before introducing the mechanization of the space-stable platform system, the main coordinate frames are given first:

(a) Inertial frame (i-frame)

The inertial system (i-frame) is a right-hand orthogonal coordinate system with the origin at the geocentre. The *Xi*-axis is parallel to the intersecting line of the equatorial plane, and the local meridian plane at initial time (*t* = 0), pointing outside from the geocentre; the *Zi*-axis coincides with the Earth’s spin axis.

(b) Earth frame (e-frame)

The Earth system (e-frame) is also a right-hand orthogonal coordinate system with the origin at the geocentre. The three axes are fixed with the Earth; the *Xe*-axis is parallel to the intersecting line of the local meridian plane at initial time (*t* = 0) and the equatorial plane, pointing outside from the centre of the Earth; the *Ze*-axis coincides with the Earth’s spin axis.

(c) Geographic frame (n-frame)

In this paper, the geographic frame is defined with the north-, east-, down-pointing axes: the origin is the centroid of the vehicle; the *Xn*-axis, also known as the north (*N*) axis, is perpendicular to the meridian plane which contains the vehicle, directly toward the north; the *Yn*-axis, also known as the east (*E*) axis, points to geodetic east; and the *Zn*-axis, also known as the down (*D*) axis, is directly downward along the geocentric vertical through the vehicle.

**Figure 2:**
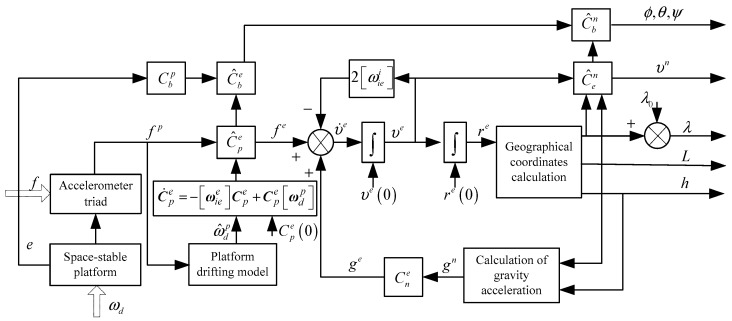
Mechanization of space-stable platform Inertial Navigation System (INS).

Since the space-stable platform does not torque on the gyroscope, the platform frame (p-frame) coincides with the i-frame, and the mechanization can take either the i-frame or the e-frame as the navigation calculation frame. In this paper, the e-frame is adopted, and the mechanization diagram of the space-stable platform INS is shown in Figure 2 [30,31,32,33,34,35,36].

In the e-frame, the navigation calculation equation of the INS is:

(5) $$\begin{array}{l} {\dot{r}}^{e}=v^{e}\\ {\dot{v}}^{e}=C_{p}^{e}f^{p}-2\left[\omega_{ie}^{e}\right]v^{e}+g^{e}\\ {\dot{C}}_{p}^{e}=-\left[\omega_{ie}^{e}\right]C_{p}^{e}+C_{p}^{e}\left[\omega_{d}^{p}\right] \end{array}$$

where, [•] stands for the skew symmetric matrix of a small angle vector, for example, if $\alpha ={\left[\begin{array}{ccc} \alpha_{1} & \alpha_{2} & \alpha_{3} \end{array}\right]}^{T}$, then $\left[\alpha \right]=\left[\begin{array}{ccc} 0 & -\alpha_{3} & \alpha_{2}\\ \alpha_{3} & 0 & -\alpha_{1}\\ -\alpha_{2} & \alpha_{1} & 0 \end{array}\right]$; $r^{e}$ is the position vector from the center of earth to the vehicle in e-frame; $v^{e}$ is the velocity vector in e-frame; $C_{p}^{e}$ is the transition matrix from p-frame to e-frame; $f^{p}$ is the specific force measured by the accelerometer triad in p-frame; $\omega_{ie}^{e}$ is the Earth's rotation angular velocity vector in e-frame; $g^{e}$ is the gravitational acceleration vector in e-frame; $\omega_{d}^{p}$ is the angular velocity of comagnetometer drift in p-frame.

The position (longitude *λ*, latitude *L* and altitude *h*) in n-frame can be iterated using the position vector $r^{e}={\left[\begin{array}{ccc} r_{x}^{e} & r_{y}^{e} & r_{z}^{e} \end{array}\right]}^{T}$ in e-frame:

(6) $$\left[\begin{array}{c} L\\ \lambda \\ h \end{array}\right]=\left[\begin{array}{c} \arctan\left(\frac{r_{z}^{e}}{\sqrt{{\left(r_{x}^{e}\right)}^{2}+{\left(r_{y}^{e}\right)}^{2}}}\left(1+\frac{e^{2}R_{N}\sin L}{r_{z}^{e}}\right)\right)\\ \lambda_{0}+\arctan\left(\frac{r_{y}^{e}}{r_{x}^{e}}\right)\\ \frac{{\left(r_{x}^{e}\right)}^{2}+{\left(r_{y}^{e}\right)}^{2}}{\cos L}-R_{N} \end{array}\right],$$

where *e* is the eccentricity of the Earth; *R_N_* is the radius of curvature of the prime vertical plane, *λ*_0_ is the initial longitude.

The velocity vector in the n-frame can be solved using the velocity vector $v^{e}$ in the e-frame:

(7) $$v^{n}=C_{e}^{n}v^{e},$$

where $C_{e}^{n}=\left[\begin{array}{ccc} -\cos\lambda \sin L & -\sin\lambda \sin L & \cos L\\ -\sin\lambda & \cos\lambda & 0\\ -\cos\lambda \cos L & -\sin\lambda \cos L & -\sin L \end{array}\right]$.

Then the attitude matrix $C_{b}^{n}$ is calculated using the matrix $C_{b}^{p}$ measured using platform frame angle and the matrix $C_{p}^{e}$ from p-frame to e-frame:

(8) $$C_{b}^{n}=C_{e}^{n}C_{p}^{e}C_{b}^{p}.$$

Finally, the attitude can be calculated using $C_{b}^{n}$.

### 4.2. Error Model of the System

According to the space-stable mechanization equation, the error equation of the comagnetometer INS can be derived by the Ψ-angle error model as:

(9) $$\begin{array}{l} \delta {\dot{r}}^{e}=\delta v^{e}\\ \delta {\dot{v}}^{e}=-2\left[\omega_{ie}^{e}\right]\delta v^{e}-\left[\omega_{ie}^{e}\right]\left[\omega_{ie}^{e}\right]\delta r^{e}+\left[f^{e}\right]\Psi^{e}+C_{p}^{e}\delta f^{p}\\ {\dot{\Psi }}^{e}=-\left[\omega_{ie}^{e}\right]\Psi^{e}+C_{p}^{e}\delta \omega_{d}^{p} \end{array},$$

where, $\delta r^{e}$ is the position error vector of the vehicle in e-frame; $\delta v^{e}$ is the velocity error vector relative to the Earth in e-frame; $\Psi^{e}={\left[\begin{array}{ccc} \varphi_{x}^{e} & \varphi_{y}^{e} & \varphi_{z}^{e} \end{array}\right]}^{T}$ presents the drift error angle of the p-frame relative to the calculation frame (e-frame); $\delta f^{p}$ stands for measurement error vector of the accelerometer triad in p-frame; and $\delta \omega_{d}^{p}$ is the angular velocity error of comagnetometer drift.

Multiplying $C_{e}^{n}$ by both sides of (9), and considering the displacement angular velocity $\rho^{n}$ from n-frame to e-frame, the Ψ-angle error equation can be transformed to n-frame:

(10) $$\begin{array}{l} \delta {\dot{r}}^{n}=\delta v^{n}-\left[\rho^{n}\right]\delta r^{n}\\ \delta {\dot{v}}^{n}=-\left(2\left[\omega_{ie}^{n}\right]+\left[\rho^{n}\right]\right)\delta v^{n}-\left[\omega_{ie}^{n}\right]\left[\omega_{ie}^{n}\right]\delta r^{n}+\left[f^{n}\right]\Psi^{n}+\delta f^{n}\\ {\dot{\Psi }}^{n}=-\left[\omega_{ie}^{n}+\omega_{en}^{n}\right]\Psi^{n}+C_{p}^{n}\delta \omega_{d}^{p} \end{array},$$

where, $\rho^{n}={\left[\begin{array}{ccc} \frac{v_{E}}{R_{N}+h} & -\frac{v_{N}}{R_{M}+h} & -\frac{v_{E}}{R_{N}+h}\tan L \end{array}\right]}^{T}$, $R_{M}$ is radius of curvature in meridian; $R_{N}$ is radius of curvature in prime vertical; *h* is the altitude; $v_{E}$ and $v_{N}$ are the velocity components in east- and north- direction.

### 4.3. Error Propagation Rule

Simplifying (10), and omitting the cross-coupling items of the vertical and horizontal loops, the position error equation of horizontal loop is [31]:

(11) $$\begin{array}{l} \delta {\ddot{r}}_{N}=-2\omega_{ie}\sin L\delta {\dot{r}}_{E}-\left(\omega_{s}^{2}-\omega_{ie}^{2}\sin^{2}L\right)\delta r_{N}+g\varphi_{y}^{e}+\delta f_{N}\\ \delta {\ddot{r}}_{E}=2\omega_{ie}\sin L\delta {\dot{r}}_{N}-\left(\omega_{s}^{2}-\omega_{ie}^{2}\right)\delta r_{E}-g\left(-\varphi_{x}^{e}\sin L+\varphi_{z}^{e}\cos L\right)+\delta f_{E} \end{array},$$

where $\omega_{s}=\sqrt{g/R}$ is the Schuler oscillating angular frequency.

It can be seen that the space-stable platform INS has similar Schuler, Foucault and Earth oscillation with the north-slaved local-level INS.

Because the Schuler and Foucault oscillation errors are all restrained by horizontal damping in long-term navigation, they are omitted from the equation solution for simplification. Assuming that the initial attitude error is $\Psi_{0}^{e}={\left[\begin{array}{ccc} \varphi_{x0}^{e} & \varphi_{y0}^{e} & \varphi_{z0}^{e} \end{array}\right]}^{T}$, the comagnetometer measurement error is $\delta \omega_{d}^{p}={\left[\begin{array}{ccc} \delta \omega_{x} & \delta \omega_{y} & \delta \omega_{z} \end{array}\right]}^{T}$, the accelerometer measurement error is $\delta f^{p}={\left[\begin{array}{ccc} \delta f_{x} & \delta f_{y} & \delta f_{z} \end{array}\right]}^{T}$, the time domain solution of the space-stable platform INS’s position error is:

(12) $$\begin{array}{l} \delta L=-\varphi_{x0}^{e}\sin\left(\omega_{ie}t\right)+\varphi_{y0}^{e}\cos\left(\omega_{ie}t\right)-\delta \omega_{x}t\sin\left(\omega_{ie}t\right)+\delta \omega_{y}t\cos\left(\omega_{ie}t\right)\\ \;+\delta f_{x}\sin L\cos\left(\omega_{ie}t\right)/g+\delta f_{y}\sin L\sin\left(\omega_{ie}t\right)/g-\delta f_{z}\cos L/g\\ \delta \lambda =\varphi_{x0}^{e}\cos\left(\omega_{ie}t\right)\sin L+\varphi_{y0}^{e}\sin\left(\omega_{ie}t\right)\sin L-\varphi_{z0}^{e}+\delta \omega_{x}t\cos\left(\omega_{ie}t\right)\sin L\\ \;+\delta \omega_{y}t\sin\left(\omega_{ie}t\right)\sin L-\delta \omega_{z}t\cos L+\delta f_{x}\sin\left(\omega_{ie}t\right)/g-\delta f_{y}\cos\left(\omega_{ie}t\right)/g \end{array}$$

Then the main error source of the space-stable platform INS in long-term navigation is analysed from (12). Position error-related items caused by initial attitude error and accelerometer bias are both bounded errors, while the position error caused by bias infinitely increases with the navigation time increasing. Specifically, the *z*-axis bias causes a longitude error linearly increasing with time, which is similar to the one caused by up- and north-axis biases in the north-slaved, local-level INS. The *x*- and *y*-axis biases cause longitude and latitude errors oscillating with the Earth period, and their amplitudes increase with the navigation time linearly; this is an error characteristic that has never been identified in the north-slaved, local-level INS. It is because the inertial instruments in the two types of INSs have different orientations relative to the Earth’s gravitational field.

According to (4), the measurement error of the comagnetometer in the INS is:

(13) $$\delta \omega_{d}^{p}=\left(\delta S+M\right)\omega_{ie}^{e}+\varepsilon .$$

where, $\delta S=\left[\begin{array}{ccc} \delta S_{x} & 0 & 0\\ 0 & \delta S_{y} & 0\\ 0 & 0 & \delta S_{z} \end{array}\right]$ is the scale factor error matrix, $M=\left[\begin{array}{ccc} 0 & \alpha_{xy} & \alpha_{xz}\\ \alpha_{yx} & 0 & \alpha_{yz}\\ \alpha_{zx} & \alpha_{zy} & 0 \end{array}\right]$ is the misalignment matrix and $\varepsilon ={\left[\begin{array}{ccc} \varepsilon_{x} & \varepsilon_{y} & \varepsilon_{z} \end{array}\right]}^{T}$ is the bias matrix of comagnetometer.

In e-frame, the Earth's rotation angular velocity is $\omega_{ie}^{e}={\left[\begin{array}{ccc} 0 & 0 & \omega_{ie} \end{array}\right]}^{T}$. With (12) and (13), we can extract the divergent errors as:

(14) $$\begin{array}{l} \delta L=-\left(\alpha_{xz}\omega_{ie}+\varepsilon_{x}\right)t\sin\left(\omega_{ie}t\right)+\left(\alpha_{yz}\omega_{ie}+\varepsilon_{y}\right)t\cos\left(\omega_{ie}t\right)\\ \delta \lambda =\left(\alpha_{xz}\omega_{ie}+\varepsilon_{x}\right)t\cos\left(\omega_{ie}t\right)\sin L+\left(\alpha_{yz}\omega_{ie}+\varepsilon_{y}\right)t\sin\left(\omega_{ie}t\right)\sin L-\left(\delta S_{z}\omega_{ie}+\varepsilon_{z}\right)t\cos L \end{array}$$

It can be seen from (14) that the main error source of the comagnetometer space-stable platform INS in long-term navigation includes the biases of the three gyroscopes, the scale factor error of the *z*-axis gyroscope, and the misalignment of the *x*- and *y*-axis gyroscope in the *z*-direction. The difference from a north-slaved, local-level INS is that the latitude error of the space-stable INS can also increase unboundedly with time in long-term navigation, as does the amplitude of the Earth periodical position error.

The divergent position errors caused by comagnetometer errors are summarised in Table 1.

## 5. Simulation Tests

### 5.1. Numerical Simulation of the Mathematical Model

**Figure 3:**
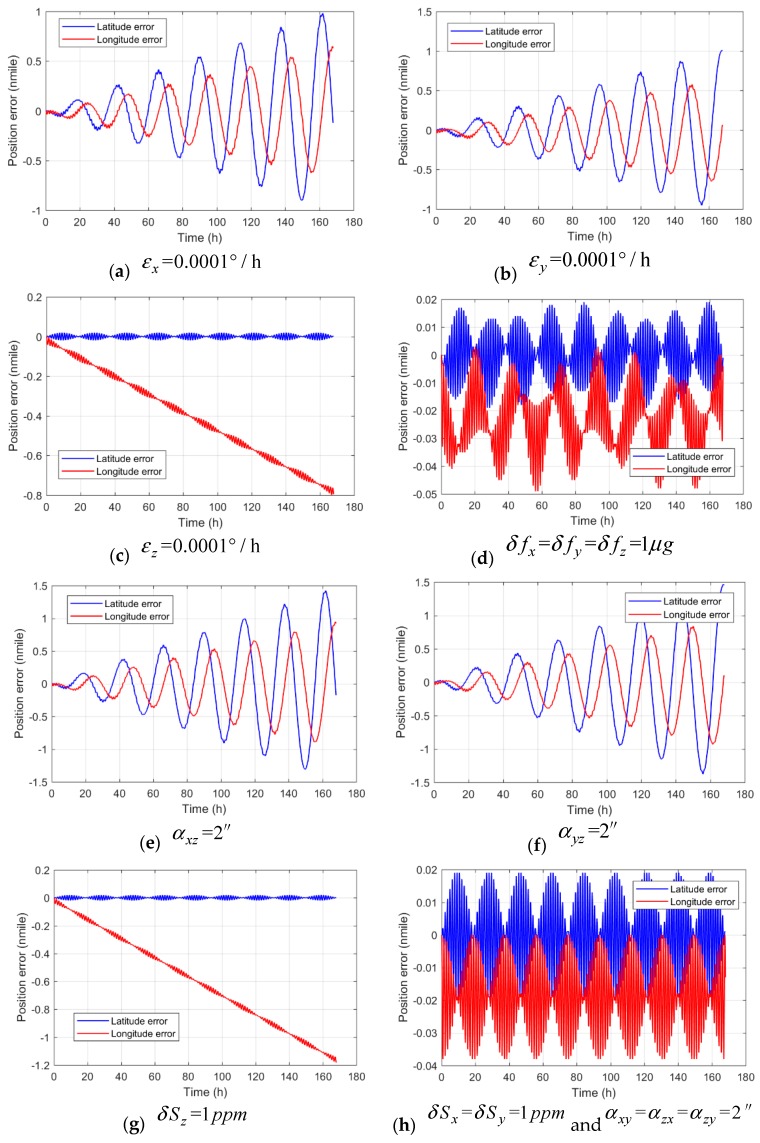
Single error test result in a numerical simulation of the mathematical model.

To verify the results of error propagation analysis, a numerical simulation of the mathematical model for each error term is carried out. By setting *x*-, *y*- and *z*-axis biases as 0.0001°/h, the accelerometer biases as 1 μg, the scale factor error as 1 ppm, and the misalignment as 2″ respectively, a 168 h (7 days) long-term navigation simulation is performed. The single error test results are presented in Figure 3a–h. It can be seen from the figures that the *x*- and *y*-axis biases cause longitude and latitude errors oscillating with the Earth period, and that the oscillations amplify with the navigation time linearly; the *z*-axis bias causes a longitude error linearly increasing with time, which is not affected by the Earth period. The accelerometer biases do not cause any unbounded error increasing with time. The scale factor error of the *z*-axis causes a longitude error similar to that of the *z*-axis bias; the misalignment due to the *x*- and *y*-axis non-orthogonal with the *z*-axis causes longitude and latitude errors, similar to that of the *x*- and *y*-axis bias. The simulation result verified the error propagation and its period analysis result found in Section 4.3.

The max position errors of the numerical simulation are shown in Table 2. It can be seen that the simulation results are consistent with the calculation result by taking simulation conditions into the error expressions in Table 1, which proves the analysis result about the error propagation rule. Thus, it can be inferred that the three-axis biases, the scale factor of the *z*-axis and the misalignment due to the *x*- and *y*-axis non-orthogonal with the *z*-axis are the main error source in long-term navigation of the comagnetometer INS.

### 5.2. Numerical Simulation Based on Semi-Physical Data

**Figure 4:**
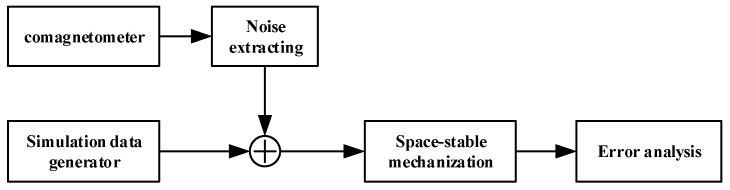
Numerical simulation based on semi-physical data.

**Figure 5:**
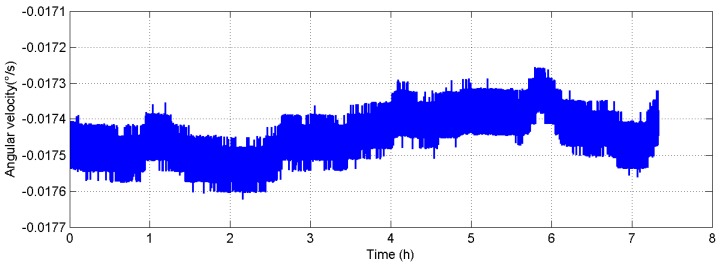
Raw data of the comagnetometer.

The design of the semi-physical simulation test is shown in Figure 4. The random error is extracted from the raw data of the comagnetometer prototype. A group of 7-h comagnetometer raw data with a sampling frequency of 200 Hz is shown in Figure 5. The simulation data is generated by superimposing the numerical simulation data of the mathematical model and the real random error. Taking this semi-physical data as input, the off-line simulation of the system is performed.

**Figure 6:**
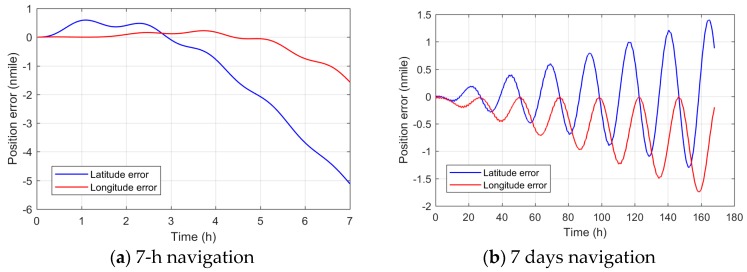
Position error in numerical simulation based on semi-physical data.

First, add the comagnetometer random error with the mean square error of 0.01°/h into the simulation data. The position error result of the 7-h navigation result is shown in Figure 6a. The position accuracy can reach 1 nmile/4 h, which preliminarily proves the feasibility of using the comagnetometer in a space-stable platform INS. Then, decrease the comagnetometer random error to the mean square error of 0.0001°/h proportionally and recycle the raw data. The position error result of 168 h (7 days) navigation is shown in Figure 6b. The position accuracy can reach 1 nmile/7 days, which preliminarily proves the potential of comagnetometer use in long-term navigation.

## 6. Conclusions and Discussion

In this paper, a space-stable navigation scheme, as well as its mechanization, is proposed according to the comagnetometer’s application characteristic. By establishing the error model of the space-stable system and analysing its error propagation rule, some useful conclusions are derived about theoretical positioning accuracy in long-term independent navigation using the comagnetometer INS: The *x*- and *y*-axis biases and the misalignment due to the *x*- and *y*-axis non-orthogonal with the *z*-axis cause longitude and latitude errors oscillating with the Earth period, and amplify linearly with navigation time; the bias and the scale factor error of the *z*-axis causes longitude error increasing with time. A numerical simulation of the mathematical model results of each single error verified the theoretical analysis result of the system’s error propagation. The three-axis biases, the scale factor of the *z*-axis and the misalignment of the *x*- and *y*-axis non-orthogonal with the *z*-axis are the main error source in long-term navigation of the comagnetometer INS. For an INS with the position accuracy of 1 nmile/7 days, the bias, the scale factor and the misalignment of the INS should meet the performance specification requirement with an accuracy better than 0.0001°/h, 1 ppm and 2″, respectively. A numerical simulation of the semi-physical data proves that the navigation scheme proposed in this paper is feasible.

This paper provides a theoretical basis for comagnetometer INS design and also presents the requirements of the comagnetometer prototype. The bandwidth of the angular velocity measurement mode and the development of an angle measurement mode represent key points for future research. 

## Figures and Tables

**Table 1:** Position errors caused by comagnetometer errors.

Error Term	Latitude Error	Longitude Error
$\varepsilon_{x}$	$-t\sin\left(\omega_{ie}t\right)$	$t\cos\left(\omega_{ie}t\right)\sin L$
$\varepsilon_{y}$	$t\cos\left(\omega_{ie}t\right)$	$t\sin\left(\omega_{ie}t\right)\sin L$
$\varepsilon_{z}$	-	−tcosL
$\alpha_{xz}$	$-\omega_{ie}t\sin\left(\omega_{ie}t\right)$	$\omega_{ie}t\cos\left(\omega_{ie}t\right)\sin L$
$\alpha_{yz}$	$\omega_{ie}t\cos\left(\omega_{ie}t\right)$	$\omega_{ie}t\sin\left(\omega_{ie}t\right)\sin L$
$\delta S_{z}$	-	$-\omega_{ie}t\cos L$

**Table 2:** Max position error in numerical simulation of the mathematical model.

Error Term	Max Latitude Error	Max Longitude Error
$\varepsilon_{x}=0.0001^{\circ }/h$	0.98 nmile	0.65 nmile
$\varepsilon_{y}=0.0001^{\circ }/h$	1.01 nmile	0.65 nmile
$\varepsilon_{z}=0.0001^{\circ }/h$	0.02 nmile	0.8 nmile
$\alpha_{xz}=2\prime \prime$	1.41 nmile	0.95 nmile
$\alpha_{yz}=2\prime \prime$	1.45 nmile	0.92 nmile
$\delta S_{z}=1ppm$	0.02 nmile	1.18 nmile
